# Black flesh disorder in mango: a chilling injury symptom increased by early harvest and low storage temperature and reduced by 1‐methylcyclopropene

**DOI:** 10.1002/jsfa.70681

**Published:** 2026-04-26

**Authors:** Bruna Parente de Carvalho Pires, João Claudio Vilvert, Nilo Ricardo Corrêa de Mello Júnior, Sandy Raiele Sena Monteiro, Mikaele de Souza Santos, Ester Silva Regis, Jackson Teixeira Lobo, Jeffrey K. Brecht, Sergio Tonetto de Freitas

**Affiliations:** ^1^ Brazilian Agricultural Research Corporation, Tropical Semi‐Arid Embrapa Petrolina Brazil; ^2^ Faculty of Agricultural and Veterinary Sciences São Paulo State University Jaboticabal Brazil; ^3^ Graduate Program in Agronomy Federal University of Vale do São Francisco Petrolina Brazil; ^4^ AgroFresh Brasil Ltda São Paulo Brazil; ^5^ Horticultural Sciences Department Institute of Food and Agricultural Sciences, University of Florida Gainesville FL USA

**Keywords:** *Mangifera indica*, 1‐MCP, chilling injury, internal disorder, antioxidant activity, phenolic compounds

## Abstract

**BACKGROUND:**

Black flesh (BF) is an internal disorder in mango, characterized by the development of dark brown to black pigmentation in the inner mesocarp tissue during storage or transport. This study investigated the effects of harvest maturity, low storage or low transport temperatures, and 1‐methylcyclopropene (1‐MCP) on mango fruit susceptibility to BF. In the first experiment, ‘Tommy Atkins’, ‘Kent’, and ‘Palmer’ mangoes were harvested at two maturity stages and BF incidence was assessed after 30 days’ storage at 8, 10, or 12.5 °C plus 7 days of shelf life at 20 °C. In the second experiment, ‘Tommy Atkins’ and ‘Keitt’ mangoes were also treated at harvest with the ethylene action inhibitor 1‐MCP (0–800 nL L^−1^) and stored at 9 °C for 45 days, followed by 7 days of shelf life at 20 °C. The most effective 1‐MCP treatment concentration (200 nL L^−1^) was then validated on mangoes for reduction of BF incidence and severity.

**RESULTS:**

The incidence of BF increased with early harvest and lower storage temperatures (≤10 °C), but it was not observed at 12.5 °C. 1‐Methylcyclopropene at 200 nL L^−1^ markedly reduced the incidence and severity of BF, while suppressing respiration and ethylene production. This concentration also maintained flesh firmness and antioxidant capacity, performing in a similar way to higher concentrations.

**CONCLUSION:**

Early harvest and low storage temperature increased the incidence of BF whereas 1‐MCP treatment reduced it. © 2026 The Author(s). *Journal of the Science of Food and Agriculture* published by John Wiley & Sons Ltd on behalf of Society of Chemical Industry.

## INTRODUCTION

Mango (*Mangifera indica* L.), which is cultivated in tropical and subtropical regions, is commercially important worldwide due to its pleasant flavor and aroma, exotic appearance, and high nutritional value.^1^, ^2^ Brazil ranks as the sixth largest producer of mangoes worldwide, with an estimated production of 1.76 million t in 2023.^3^ The expansion of mango cultivation in the country has been driven by its high commercial value, especially in the international market, with strong demand for cultivars such as ‘Tommy Atkins’, ‘Palmer’, ‘Keitt’, and ‘Kent’ in Europe, Asia, and North America.^2^, ^4^, ^5^, ^6^

Mango is currently Brazil's primary fruit export, with the irrigated semi‐arid São Francisco Valley accounting for over 90% of national exports.^7^ Shipping mangoes to distant markets requires low temperatures to manage their rapid ripening.^8^ However, mangoes are highly sensitive to cold, which can lead to chilling injury (CI).^9^ Commonly reported CI symptoms include aroma loss and uneven ripening, lenticel discoloration, and peel browning.^10^, ^11^ Recent findings suggest that low temperatures may trigger internal flesh darkening,^12^ but no further studies have tested the effects of cold temperatures on the incidence and severity of this condition, referred to as ‘black flesh’ (BF).

Black flesh, which is also known as cutting black, black spot, flesh browning, *corte negro*, or *pulpa negra*, is an internal disorder of mango fruit characterized by a diffuse flesh discoloration around the seed, ranging from brown to black, possibly resulting from cell wall and membrane degradation.^12^, ^13^, ^14^ It has been recently reported to be one of the most important mango internal disorders, which can affect up to 100% of the fruit during storage and/or shipping under commercial conditions, representing a major concern for the mango industry.^12^, ^15^

Given reports linking BF incidence to low storage temperatures,^12^ it is important to understand the mechanisms regulating CI in order to identify factors contributing to BF. A primary CI mechanism is the reduction of the unsaturated‐to‐saturated fatty acid ratio in cellular membranes. This increases membrane rigidity and susceptibility to lipid peroxidation, triggered by reactive oxygen species (ROS) under cold stress. This process is exacerbated by early harvest and the exposure of immature fruit to low temperatures.^16^

1‐Methylcyclopropene (1‐MCP) – a gaseous cyclopropene derivative that irreversibly binds to cellular ethylene receptors^1^1 – has emerged as a potential technology for mitigating CI. The effects of 1‐MCP have been extensively investigated for extending shelf life and delaying senescence.^17^, ^18^, ^19^ Recent studies have shown that 1‐MCP has not only inhibited ethylene‐induced ripening processes, but has also improved membrane stability by scavenging ROS.^11^, ^20^, ^21^, ^22^

Reduced CI symptoms following 1‐MCP application have been documented in avocado,^23^ nectarine,^24^ and apple.^25^ In mangoes,^26^ the mitigation of external CI symptoms by 1‐MCP has been demonstrated in two cultivars. Studies have reported that 1‐MCP‐treated mangoes exhibited lower malondialdehyde (MDA) content – a byproduct of lipid peroxidation – suggesting enhanced membrane integrity via reduced oxidative stress.^27^ 1‐Methylcyclopropene therefore increased fruit resistance to chilling stress, potentially playing a role in preventing the internal browning associated with BF. However, no studies have specifically evaluated whether BF represents a form of CI or tested the effectiveness of 1‐MCP in mitigating the disorder. Based on this evidence, it was hypothesized that BF incidence could be increased in mango fruit using factors that increase fruit susceptibility to CI, such as early harvest and low storage temperature, and reduced with treatments that reduce susceptibility, such as 1‐MCP treatment.

The aims of this study were therefore to: (1) investigate the relationship between maturity stage, storage temperature, and BF incidence in mangoes; (2) determine the optimal 1‐MCP concentration for alleviating BF; and (3) validate the effectiveness of 1‐MCP in controlling BF while evaluating its influence on physiological attributes, quality parameters, and antioxidant metabolism. The storage durations employed (28–45 days) are consistent with commercial mango international transport, storage, and distribution times.

## MATERIALS AND METHODS

### Plant material and experimental design

The experiments were conducted with mangoes harvested from commercial orchards in the São Francisco Valley, Petrolina, PE, Brazil. The orchards were selected based on their history of high BF incidence. The region has a semi‐arid (BSh) climate, according to the Köppen classification.^28^ After harvest, fruit were transported to the Postharvest Physiology and Technology Laboratory at Embrapa, Petrolina, PE, Brazil, for treatment application and subsequent assessment. Fruit were selected for uniform size and absence of defects, visible injuries, and diseases.

**Figure 1:**
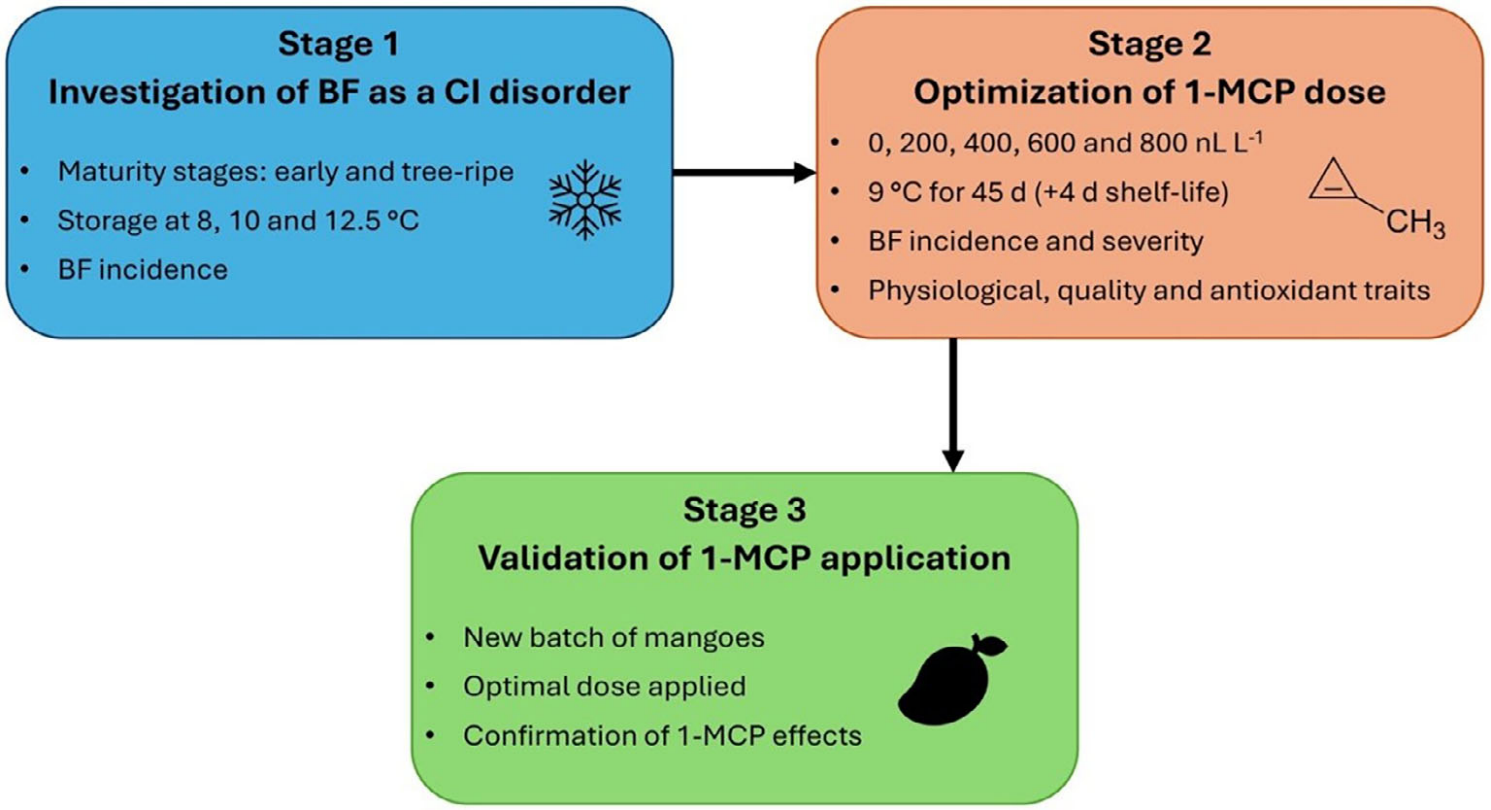
Overview of the experimental stages of the study.

The study comprised three experimental stages, conducted over 3 years, as summarized in Fig. 1. In the first stage, the incidence and severity of BF were evaluated in mangoes harvested at two maturity stages (early and tree ripe) and stored at three temperatures, to determine the effect of harvest maturity and storage temperature on BF incidence and severity, a possible CI disorder. In the second stage, different postharvest 1‐MCP (AgroFresh Brasil Ltda, São Paulo, Brazil) doses were tested to alleviate BF in early maturity stage mangoes and assess their effects on fruit quality and antioxidant metabolism, with the goal of identifying an optimal dose. In the third stage, the optimal 1‐MCP dose was applied to a new batch of early maturity stage mangoes as a validation step. The three stages are described in detail below.

### Experiment 1: effect of maturity stage and storage temperature on BF incidence

A total of 600 ‘Tommy Atkins’, 600 ‘Kent’, and 600 ‘Palmer’ mangoes were harvested at two maturity stages, corresponding to stage 1 (early maturity; i.e. physiologically mature but prior to onset of ripening) and Stage 3 (advanced, ‘tree‐ripe’ maturity; approximately one‐half ripe based on internal color development) on a standard commercial mango 1–5 maturity/ripeness scale.^29^ At the early maturity stage, ‘Tommy Atkins’ mangoes showed flesh firmness of 84.2 N, soluble solid content (SSC) of 10.7%, and titratable acidity (TA) of 1.03%. At this stage, ‘Palmer’ mangoes had a firmness of 115.9 N, SSC of 10.3%, and TA of 1.0%, whereas ‘Kent’ mangoes showed flesh firmness of 89.4 N, SSC of 9.6%, and TA of 1.1%. At the advanced maturity stage, ‘Tommy Atkins’ mangoes showed flesh firmness of 47.0 N, SSC of 12.6%, and TA of 0.67%, whereas ‘Palmer’ mangoes had a firmness of 100.8 N, SSC of 12.3%, and TA of 0.69%, and ‘Kent’ mangoes showed flesh firmness of 56.9 N, SSC of 12.3%, and TA of 0.58%.

Fruit from each cultivar and maturity stage were stored at 8 °C, 10 °C, or 12.5 °C with relative humidity of 90% to 95% for 30 days. These temperatures have been reported to result in high, medium, and low mango susceptibility to external CI symptoms during storage.^14^, ^30^, ^31^ A previous report also suggested that storage temperatures above 10 °C may reduce BF incidence in mangoes.^12^ Each treatment (cultivar × maturity stage × temperature) consisted of 100 fruit, divided into four replications of 25 fruit each. After cold storage, fruit were kept at 20 °C for 7 days to simulate shelf life conditions, at which point BF incidence was assessed. Incidence was calculated as the percentage of fruit with visual BF symptoms in each sample.

### Experiment 2: effect of 1‐MCP application on BF and fruit quality

A total of 540 ‘Tommy Atkins’ and 540 ‘Keitt’ mangoes were harvested at earlier maturity stages (1, 2).^29^ A subsample of 20 fruit per cultivar was analyzed at harvest to determine initial physicochemical quality. The remaining fruit were randomly assigned to five treatment groups, with four replicates of 104 fruit each.

The treatments were 0, 200, 400, 600, and 800 nL L^−1^ 1‐methylcyclopropene (1‐MCP) (AgroFresh Brasil Ltda, São Paulo, Brazil) (SmartFresh). Fruit were placed in sealed 120 L containers, and the appropriate amounts of 1‐MCP were dissolved in 25 mL of water and added to the containers for 24 h gas exposure at 9 °C. Following treatment, fruit were stored at 9 °C with relative humidity of 90% to 95% for 45 days. After this period, half of the fruit were analyzed immediately and the other half were held at 20 °C for 7 days to simulate shelf life. After both periods, fruit were evaluated for BF incidence and severity, physiological traits (respiration, ethylene production, and flesh firmness), physicochemical traits (peel and flesh color, soluble solids, and titratable acidity), and antioxidant metabolism (phenolic compounds and antioxidant activity by 2,2′‐azino‐bis(3‐ethylbenzothiazoline‐6‐sulfonic acid) (Sigma‐Aldrich Brasil Ltda., São Paulo, Brazil) (ABTS) and 2,2‐diphenyl‐1‐picrylhydrazyl (Sigma‐Aldrich Brasil Ltda., São Paulo, Brazil) (DPPH) assays), as described below.

#### Black flesh incidence and severity

Fruit were sliced longitudinally on both sides, parallel to and near the flat side of the stone, to expose the flesh to evaluate BF incidence and severity. Incidence (%) was calculated as described above. Severity (%) was assessed visually only in BF‐affected fruit by estimating the percentage of flesh area exhibiting symptoms. These values were then averaged across all symptomatic fruit within each sample.

#### Physiological parameters

Respiration rate and ethylene production were measured using a portable gas analyzer (model F‐950; Felix Instruments, WA, USA) in a static system with a 1 h chamber sealing period. Flesh firmness was determined as the bioyield point with a texture analyzer (model TA.XTplus; Stable Micro Systems, Godalming, UK) fitted with a 6 mm cylindrical probe used to penetrate the fullest area of the cheek after removal of a small patch of peel. Firmness results were expressed in N as the force required to penetrate 10 mm into the flesh.

#### Physicochemical quality parameters

Peel and flesh color were measured with a digital colorimeter (model CR‐400; Minolta, Tokyo, Japan) using the CIE L*a*b* color space and expressed as hue angle (0/360° = red, 90° = yellow, 180° = green, and 270° = blue). The peel ground color was measured while avoiding areas with red blush. Flesh color was measured on the inner side of each fruit half. Measurements were obtained by slicing the fruit longitudinally, parallel and close to the flat surface of the stone. The SSC was measured in juice extracted from the flesh using a handheld digital refractometer (model PAL‐1; Atago, Tokyo, Japan) and expressed as a percentage. The TA was determined from 1.0 g of expressed juice diluted in 50 mL of distilled water, which was titrated with 0.1mol L^−1^ NaOH to pH 8.1, using an automatic titrator (model Titrino Plus 848; Metrohm, São Paulo, Brazil), and expressed as percentage citric acid equivalents.

#### Total phenolic content and antioxidant activity

Total phenolic content (TPC) was determined using the Folin–Ciocâlteu method, based on the reducing capacity of phenolic compounds. For extract preparation, 8.0 g of flesh was extracted with 20 mL of 50% (Sigma‐Aldrich Brasil Ltda., São Paulo, Brazil) methanol (v/v) for 1 h, centrifuged at 10 000 × *g* for 15 min at 4 °C, and filtered. The residue was then extracted with 20 mL of 70% acetone (v/v) for 1 h under the same conditions. Both supernatants were combined in a 50 mL volumetric flask and adjusted with distilled water. The extract was used for TPC and antioxidant assays, as described below.

Aliquots of 0.5 mL of extract were diluted with distilled water to 1.0 mL, then mixed with 1.0 mL (Sigma‐Aldrich Brasil Ltda., São Paulo, Brazil) Folin–Ciocâlteu reagent, 2.0 mL of 20% (Sigma‐Aldrich Brasil Ltda., São Paulo, Brazil) sodium carbonate, and 2.0 mL distilled water. After 30 min, absorbance was measured at 700 nm. Results were expressed as mg 100 g^−1^ GAE fresh weight.^32^

Antioxidant activity (AOX) was assessed using the ABTS and DPPH radical scavenging assays.^33^, ^34^ In the ABTS assay, the ABTS•^+^ radical cation was generated by mixing equal volumes of 7 mM ABTS and 2.45 mM (Sigma‐Aldrich Brasil Ltda., São Paulo, Brazil) potassium persulfate, kept in dark conditions for 16 h at 20 °C. Before use, the solution was diluted with ethanol to an absorbance of 0.700 at 754 nm. For the assay, 30 μL of extract was added to 3.0 mL of ABTS solution. Absorbance was read at 0 and 6 min at 754 nm. Results were expressed as mM (Sigma‐Aldrich Brasil Ltda., São Paulo, Brazil) Trolox 100 g^−1^.

In the DPPH assay, a 100 μM DPPH stock solution was prepared in methanol and 0.1 mL of extract was added to 2.9 mL of 1.0 mM DPPH solution. After 30 min in dark conditions at 20 °C, absorbance was read at 517 nm. Results were expressed as mM Trolox 100 g^−1^.

### Experiment 3: validation of the optimal 1‐MCP dose

After evaluating and selecting the most efficient 1‐MCP dose (200 nL L^−1^) to control BF incidence and severity, stage 1–2 ‘Tommy Atkins’ and ‘Keitt’ mangoes were harvested again to validate the results. In the validation stage, only control (0 nL L^−1^) and 1‐MCP treatment (200 nL L^−1^) were repeated as in Experiment 2 (see above) for each cultivar. Each treatment consisted of four replications containing 12 fruit per replication. The fruit were stored and evaluated after 45 days of storage and 7 days of shelf life, as described above.

### Statistical analysis

Experiments 1 and 2 followed a completely randomized design (CRD) with four replications per treatment. A one‐way analysis of variance (ANOVA) was carried out in five steps: (1) detection of extreme outliers in the dataset; (2) assessment of normality assumption through model residuals using Q–Q plots and the Shapiro–Wilk test; (3) evaluation of homogeneity of variances by residuals versus fitted plots and Levene's test; (4) application of the F‐test to assess treatment effects; and (5) pairwise mean comparisons among treatments using the least significant difference (LSD) test (*P* < 0.05). In Experiment 3, treatment comparisons (1‐MCP at 0 × 200 nL L^−1^) were performed using non‐parametric bootstrap resampling. Bootstrap analysis was carried out with 5000 iterations, resampling with replacement from the original dataset. The mean difference between the control and treatment was calculated for each iteration. The distribution of bootstrap estimates was then used to obtain percentile‐based 95% confidence intervals for the mean difference.

## RESULTS

### Experiment 1: BF incidence in response to early harvest and storage temperature

**Figure 2:**
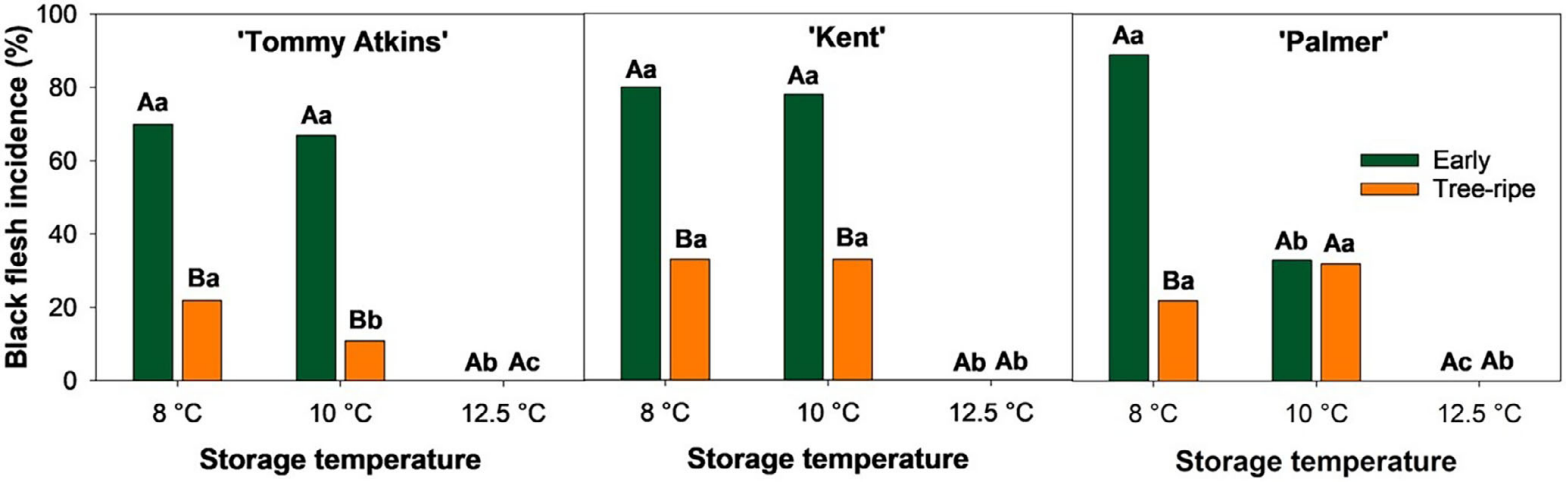
Incidence of black flesh (BF) in ‘Tommy Atkins’, ‘Kent’, and ‘Palmer’ mangoes in response to different maturity stage and storage temperature. The BF incidence was determined after 30 days of cold storage plus 7 days at 20 °C. Within each storage temperature, means followed by the same uppercase letter – and within each maturity stage by the same lowercase letter – are not significantly different according to the least significant difference (LSD) test (*P* < 0.05).

For the three mango cultivars evaluated in Experiment 1, BF incidence was influenced by fruit maturity at harvest and by storage temperature (*P* < 0.05) (Fig. 2). For ‘Tommy Atkins’ mangoes, fruit harvested at the early maturity stage exhibited a markedly higher BF incidence, particularly at lower storage temperatures, reaching 70% at 8 °C and 67% at 10 °C (Fig. 2). Conversely, no symptoms were observed at 12.5 °C, regardless of maturity stage (Fig. 2). Fruit harvested at the more advanced maturity showed a substantial reduction in BF incidence at the same temperatures, with values decreasing to 22% at 8 °C and 11% at 10 °C (Fig. 2).

For ‘Kent’ mangoes, BF symptoms were also associated strongly with low storage temperatures and harvest maturity (Fig. 2). Early harvested fruit exhibited high incidences of BF, reaching 80% at 8 °C and 78% at 10 °C, whereas no symptoms were detected at 12.5 °C (Fig. 2). Fruit harvested at the more advanced maturity showed markedly lower BF incidence, which remained at 33% at both 8 °C and 10 °C, and no symptoms were observed at 12.5 °C (Fig. 2).

For ‘Palmer’ mangoes, early harvested fruit stored at 8 °C showed the highest BF incidence (89%), which decreased to 33% at 10 °C (Fig. 2). Although late‐harvested ‘Palmer’ fruit still exhibited symptoms at 8 °C and 10 °C (22% and 32%, respectively), their incidence remained lower than in early harvested fruit stored at 8 °C (Fig. 2). For both maturity stages, no symptoms were detected at 12.5 °C (Fig. 2).

### Experiment 2: BF incidence and severity in response to 1‐MCP treatment

#### Incidence and severity of BF

**Figure 3:**
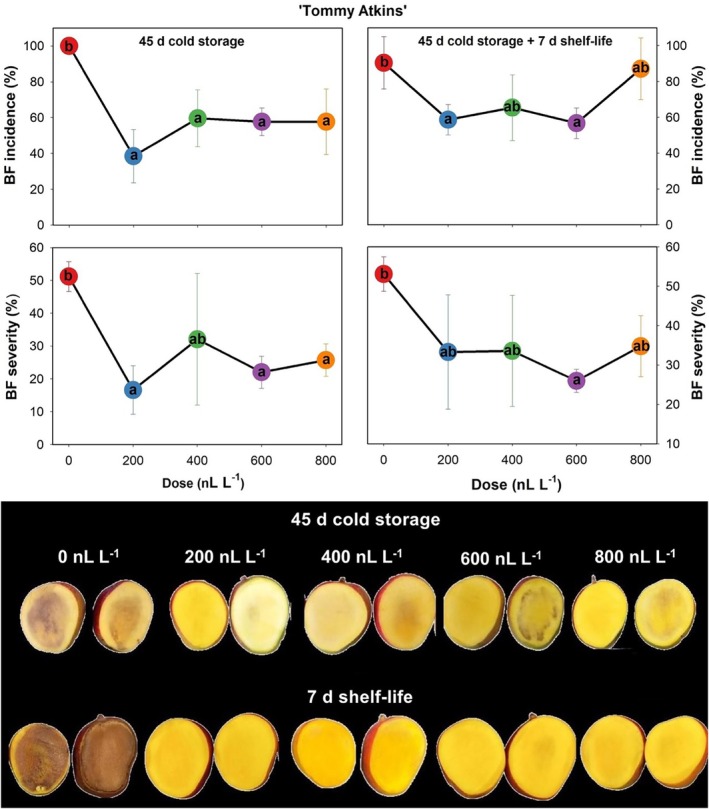
Incidence and severity of black flesh (BF) disorder in ‘Tommy Atkins’ mangoes treated with different 1‐methylcyclopropene (1‐MCP) concentrations and stored at 9 °C for 45 days followed by 7 days of shelf life at 20 °C. Data are presented as means ± standard deviations (SDs). Different letters indicate significant differences among 1‐MCP doses according to the least significant difference (LSD) test (*P* < 0.05).

**Figure 4:**
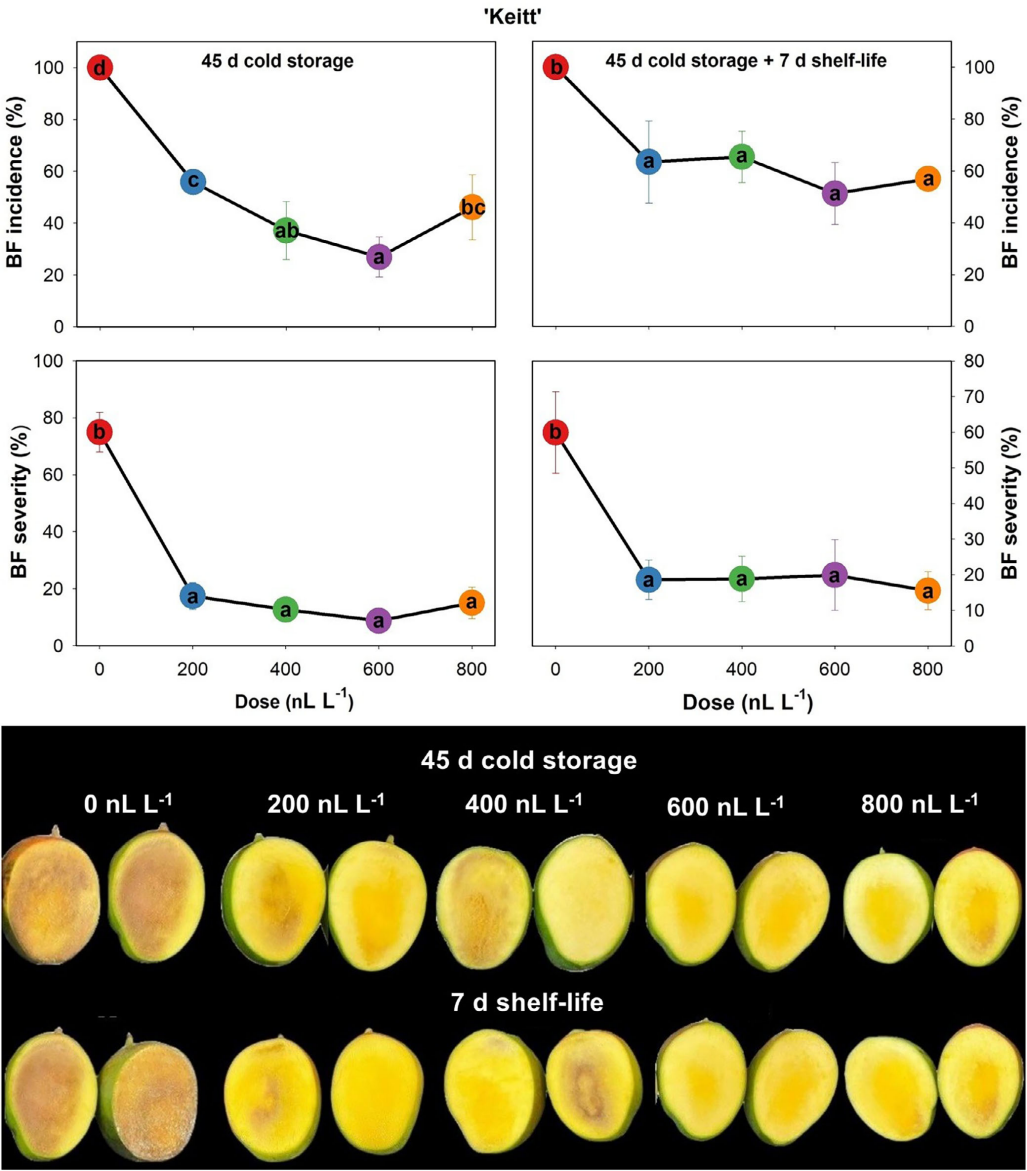
Incidence and severity of black flesh (BF) disorder in ‘Keitt’ mangoes treated with different 1‐methylcyclopropene (1‐MCP) concentrations and stored at 9 °C for 45 days followed by 7 days of shelf life at 20 °C. Data are presented as means ± standard deviations (SDs). Different letters indicate significant differences among 1‐MCP doses according to the least significant difference (LSD) test (*P* < 0.05).

Treatment with 1‐MCP significantly (*P* < 0.05) reduced the BF incidence and severity in both ‘Tommy Atkins’ and ‘Keitt’ mangoes stored at 9 °C for 45 days (Figs 3 and 4). At the end of cold storage, untreated ‘Tommy Atkins’ fruit exhibited 100% BF incidence, whereas the application of 1‐MCP (200–800 nL L^−1^) decreased the disorder to below 60% (*P* < 0.05). After 7 days of shelf life at 20 °C, untreated ‘Tommy Atkins’ fruit also showed higher BF incidence than fruit treated with 200 nL L^−1^ and 600 nL L^−1^ 1‐MCP‐treated fruit (Fig. 3). After 45 days of storage, untreated ‘Tommy Atkins’ fruit had the highest BF severity (51%), compared with fruit treated with 1‐MCP at 200, 600, and 800 nL L^−1^ (which displayed BF severity of 17% to 26%). After 7 days of shelf life, untreated ‘Tommy Atkins’ fruit maintained higher BF severity (53%) than 1‐MCP‐treated fruit at 600 nL L^−1^ (26%) (Fig. 3).

For ‘Keitt’ mangoes, the BF incidence reached 100% in untreated fruit after 45 days of cold storage, whereas 1‐MCP treatment, regardless of the concentration, decreased the disorder to below 60%. The application of 1‐MCP at 600 nL L^−1^ promoted the lowest BF incidence (27%) after cold storage. After shelf life, BF incidence was reduced from 100% in untreated fruit to below 65% in all treated fruit (Fig. 4). The severity of BF in ‘Keitt’ mangoes was also reduced by 1‐MCP treatment (*P* < 0.05), both after cold storage and after 7 days of shelf life. Untreated ‘Keitt’ mangoes exhibited high severity levels (75% after cold storage and 60% after shelf life), whereas 1‐MCP‐treated fruit showed a drastic reduction, with severity dropping to below 20% after cold storage and from 18% to 20% after shelf life. After storage and shelf life, no significant differences in severity were observed among the 1‐MCP doses tested (*P* > 0.05) (Fig. 4).

#### Physiological parameters

**Figure 5:**
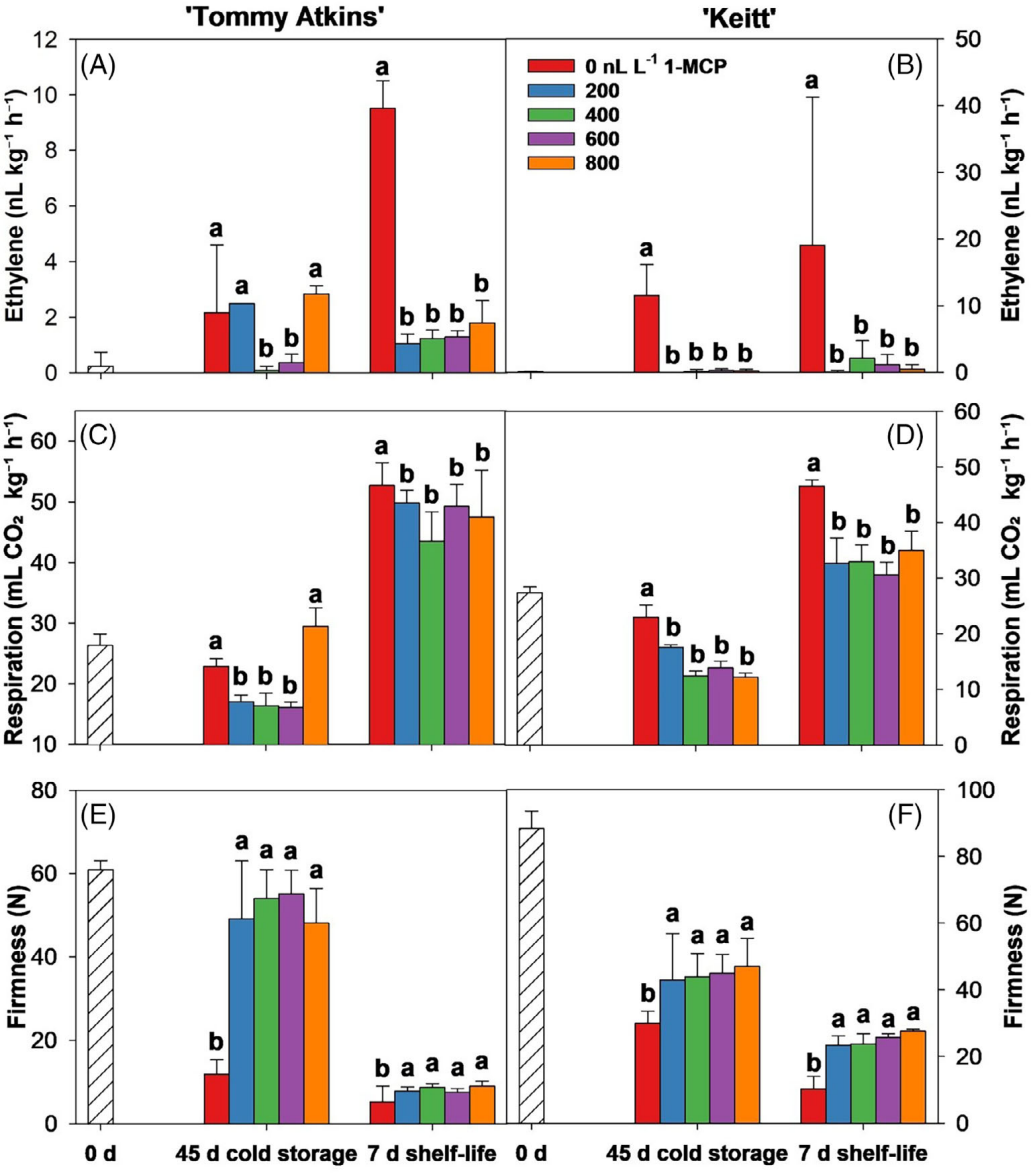
Effects of 1‐methylcyclopropene (1‐MCP) on ethylene production in ‘Tommy Atkins’ (A) and ‘Keitt’ (B), respiration rate in ‘Tommy Atkins’ (C) and ‘Keitt’ (D), and flesh firmness in ‘Tommy Atkins’ (E) and ‘Keitt’ (F) mangoes stored at 9 °C for 45 days followed by 7 days of shelf life at 20 °C. Data are presented as means ± standard deviations (SDs). Different letters indicate significant differences among 1‐MCP doses according to the least significant difference (LSD) test (*P* < 0.05).

Ethylene production was reduced markedly by 1‐MCP application in ‘Tommy Atkins’ and ‘Keitt’ mangoes, after 45 days of storage and after 7 days of shelf life (Fig. 5(A),(B)). After storage, ‘Tommy Atkins’ untreated fruit produced ethylene at a rate of 2.18 nL kg^−1^ h^−1^, whereas fruit treated with 1‐MCP at 400 nL L^−1^ (0.10 nL kg^−1^ h^−1^) or 600 nL L^−1^ (0.38 nL kg^−1^ h^−1^) exhibited lower ethylene production (Fig. 5(A)). For ‘Keitt’, untreated fruit produced ethylene at 11.58 nL kg^−1^ h^−1^, whereas 1‐MCP‐treated fruit ethylene production ranged from 0.00 to 0.35 nL kg^−1^ h^−1^, with no significant differences between 1‐MCP doses (Fig. 5(B)).

After 7 days of shelf life, ethylene production decreased from 9.52 nL kg^−1^ h^−1^ for ‘Tommy Atkins’ untreated fruit to 1.05–1.80 nL kg^−1^ h^−1^ in 1‐MCP‐treated fruit (Fig. 5(A)). A similar pattern was observed for ‘Keitt’ mangoes, with untreated fruit producing 19.13 nL kg^−1^ h^−1^ of ethylene compared with 0.13–2.18 nL kg^−1^ h^−1^ for 1‐MCP‐treated fruit (Fig. 5(B)).

The respiration rate of both cultivars was affected significantly by 1‐MCP application. Untreated ‘Tommy Atkins’ mangoes had the highest respiration rate after cold storage (22.94 mL CO_2_ kg^−1^ h^−1^), and 1‐MCP administered at 200, 400, and 600 nL L^−1^ reduced respiration to rates ranging from 16.17 to 17.45 mL CO_2_ kg^−1^ h^−1^. After 7 days of shelf life, respiration was higher in untreated fruit (51.32 mL CO_2_ kg^−1^ h^−1^) than in 1‐MCP‐treated fruit (43.50–49.90 mL CO_2_ kg^−1^ h^−1^) (Fig. 5(C)).

A similar pattern was observed with ‘Keitt’ mangoes. After cold storage, untreated fruit had a respiration rate of 23.07 mL CO_2_ kg^−1^ h^−1^, whereas 1‐MCP‐treated fruit maintained lower rates (12.30–17.67 mL CO_2_ kg^−1^ h^−1^). After shelf life, respiration reached 46.64 mL CO_2_ kg^−1^ h^−1^ in untreated fruit, whereas 1‐MCP application reduced respiration to 30.68–35.09 mL CO_2_ kg^−1^ h^−1^ (Fig. 5(D)).

The flesh firmness of both cultivars was significantly better maintained by 1‐MCP application. For ‘Tommy Atkins’ mangoes, after cold storage, untreated fruit exhibited the lowest firmness (12.09 N), whereas 1‐MCP‐treated fruit retained significantly higher values, of 48.31–55.32 N, with no significant differences among doses. After 7 days of shelf life, firmness of untreated fruit decreased to 5.43 N, whereas 1‐MCP‐treated fruit maintained higher flesh firmness values of 7.78–9.18 N (Fig. 5(E)).

A similar response was observed for ‘Keitt’ mangoes. After cold storage, untreated fruit showed firmness of 30.27 N, while 1‐MCP‐treated fruit retained flesh firmness values of 43.12–47.26 N. After shelf life, untreated fruit softened drastically to 10.48 N, whereas 1‐MCP‐treated fruit maintained significantly higher firmness of 23.65–27.88 N (Fig. 5(F)).

#### Color and physicochemical attributes

**Figure 6:**
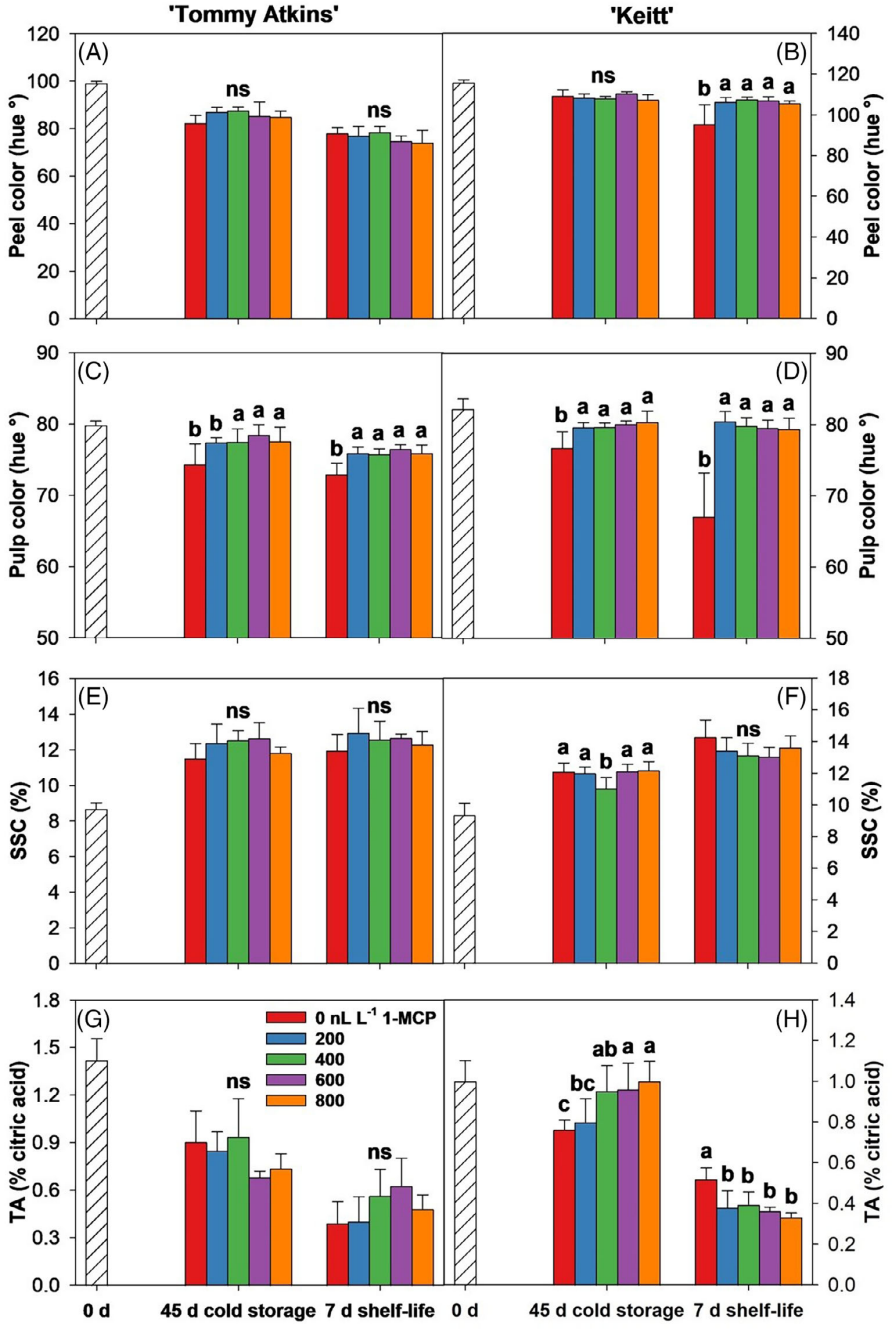
Effects of 1‐methylcyclopropene (1‐MCP) on peel color in ‘Tommy Atkins’ (A) and ‘Keitt’ (B), flesh (pulp) color in ‘Tommy Atkins’ (C) and ‘Keitt’ (D), soluble solids content (SSC) in ‘Tommy Atkins’ (E) and ‘Keitt’ (F), and titratable acidity (TA) in ‘Tommy Atkins’ (G) and ‘Keitt’ (H) mangoes stored at 9 °C for 45 days followed by 7 days of shelf life at 20 °C. Data are presented as means ± standard deviations (SDs). Different letters indicate significant differences among 1‐MCP doses according to the least significant difference (LSD) test (*P* < 0.05).

The peel color, expressed as hue angle, was not significantly affected by 1‐MCP treatments in either ‘Tommy Atkins’ or ‘Keitt’ mangoes after cold storage. For ‘Tommy Atkins’, peel hue angle was 82.2–87.5° (Fig. 6(A)), whereas for ‘Keitt’ it was 107.3–110.3° (Fig. 6(B)). After shelf life, ‘Tommy Atkins’ peel hue values were not affected by 1‐MCP, at 74.0–78.3° (Fig. 6(A)), whereas for ‘Keitt’, untreated fruit had a lower hue (95.2°), compared with 1‐MCP‐treated fruit (105.5–107.4°) (Fig. 6(B)).

Flesh color was affected significantly by 1‐MCP application for both ‘Tommy Atkins’ and ‘Keitt’ mangoes. After cold storage, untreated fruit exhibited lower hue angle values than 1‐MCP‐treated fruit, indicating more advanced ripeness in both untreated cultivars. In contrast, 1‐MCP‐treated fruit maintained greater hue angles, with values of 77.4–78.4° for ‘Tommy Atkins’ (Fig. 6(C)) and 79.6–80.3° for ‘Keitt’ mangoes (Fig. 6(D)), regardless of dose. After 7 days of shelf life, flesh color differences between untreated and 1‐MCP‐treated fruit became more pronounced. For ‘Tommy Atkins’, hue angle decreased to 72.9° in untreated fruit, and in 1‐MCP‐treated fruit it was 75.8–76.4° (Fig. 6(C)). A similar pattern was observed for ‘Keitt’, for which untreated fruit reached lower hue values (67.0°) compared with 1‐MCP‐treated fruit (79.4–80.4°) (Fig. 6(D)).

The SSC was not consistently affected by 1‐MCP application in either cultivar. For ‘Tommy Atkins’, SSC values remained stable across all treatments, ranging from 11.5% to 12.9%, both after cold storage and after 7 days of shelf life (Fig. 6(E)). For ‘Keitt’, a slight but significant reduction in SSC was observed after cold storage in fruit treated with the intermediate 1‐MCP dose (400 nL L^−1^), but this effect was not maintained after shelf life, when all treatments showed similar values (13.0% to 14.3%) (Fig. 6(F)).

The application of 1‐MCP had no effect on TA in ‘Tommy Atkins’ mangoes, regardless of whether the fruit were assessed after cold storage or after 7 days of shelf life. After cold storage, TA values ranged from 0.68 to 0.94%, and after shelf life, TA values were reduced to 0.39% and 0.63%, with no differences among treatments (Fig. 6(G)).

In contrast, significant 1‐MCP effects were observed on the TA of ‘Keitt’ mangoes. After cold storage, TA ranged from 0.76% in untreated fruit to 0.95% or 0.96% in fruit treated with 400 or 600 nL L^−1^ 1‐MCP, respectively, with intermediate values in the other treatments. After shelf life, the differences became more pronounced, with untreated fruit showing the highest acidity (0.52%), while 1‐MCP‐treated fruit had lower values, ranging from 0.33% to 0.40% (Fig. 6(H)).

#### Total phenolic content and AOX

**Figure 7:**
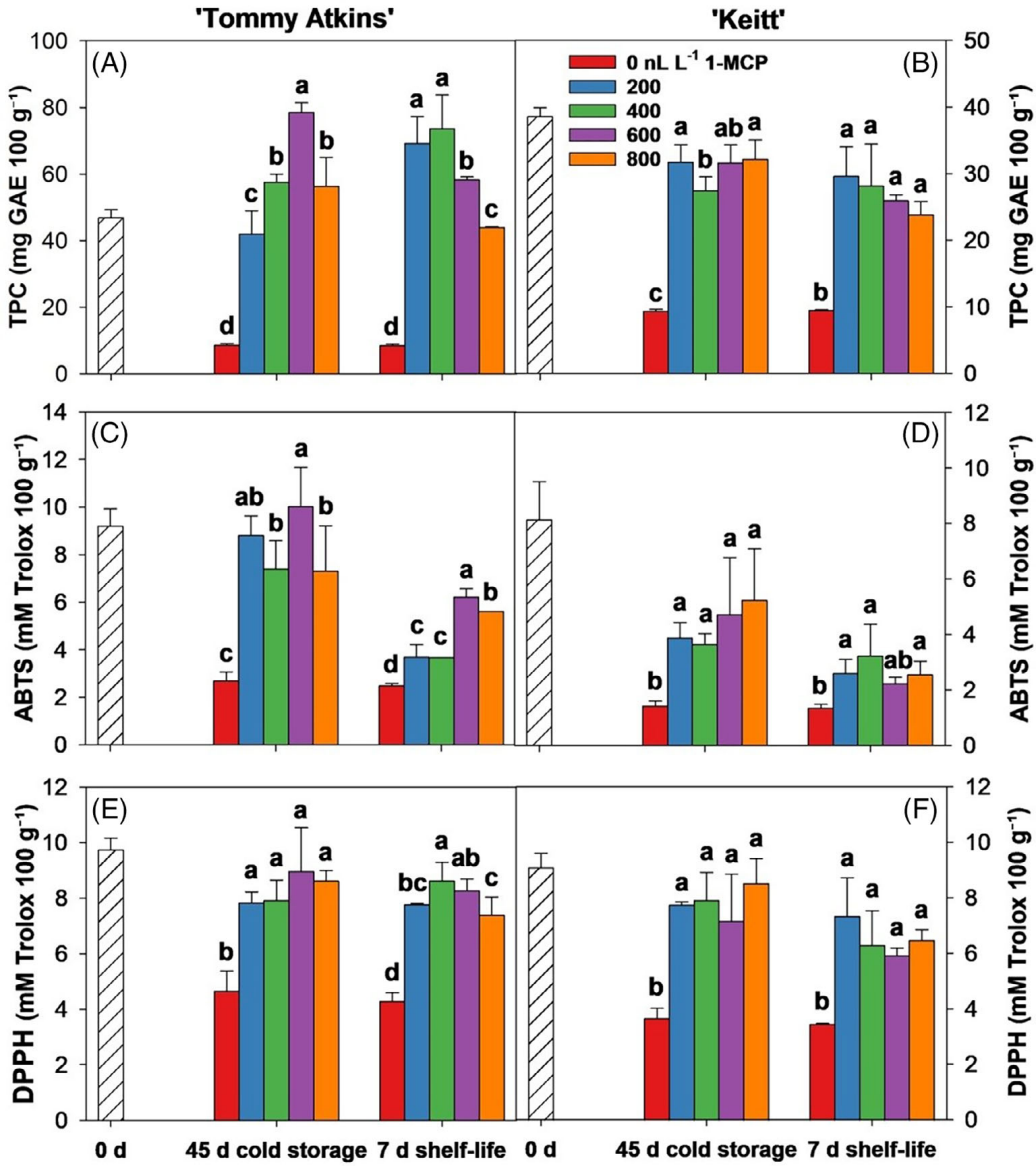
Effects of 1‐MCP on total phenolic content (TPC) in ‘Tommy Atkins’ (A) and ‘Keitt’ (B), and antioxidant activity determined by the ABTS method in ‘Tommy Atkins’ (C) and ‘Keitt’ (D) and the DPPH method in ‘Tommy Atkins’ (E) and ‘Keitt’ (F) mangoes stored at 9 °C for 45 days followed by 7 days of shelf life at 20 °C. Data are presented as means ± standard deviations (SDs). Different letters indicate significant differences among 1‐MCP doses according to the least significant difference (LSD) test (*P* < 0.05).

The TPC was influenced significantly by 1‐MCP application for both ‘Tommy Atkins’ and ‘Keitt’ mangoes. At harvest, TPC averaged 46.9 mg GAE 100 g^−1^ for ‘Tommy Atkins’. After 45 days of cold storage, untreated fruit exhibited the lowest TPC content (8.6 mg GAE 100 g^−1^), whereas 1‐MCP‐treated fruit maintained markedly higher levels, especially with the 600 nL L^−1^ dose (78.6 mg GAE 100 g^−1^). These differences were consistent after the 7 days of shelf life, when untreated fruit showed a very low TPC content (8.6 mg GAE 100 g^−1^), compared with 1‐MCP treatments. Among 1‐MCP doses, 200 nL L^−1^ (69.2 mg GAE 100 g^−1^) and 400 nL L^−1^ (73.7 mg GAE 100 g^−1^) retained higher TPC values (Fig. 7(A)).

For ‘Keitt’, the average TPC content at harvest was 38.6 mg GAE 100 g^−1^. After 45 days of cold storage, untreated fruit showed the lowest values (9.4 mg GAE 100 g^−1^), whereas 1‐MCP‐treated fruit maintained higher TPC content, ranging from 27.4 to 32.2 mg GAE 100 g^−1^. The highest TPC levels were observed with 1‐MCP doses of 800 and 200 nL L^−1^ (32.2 and 31.7 mg GAE 100 g^−1^, respectively). After 7 days of shelf life, untreated fruit presented lower TPC content (9.4 mg GAE 100 g^−1^), whereas all 1‐MCP treatments preserved significantly higher TPC content (23.8–29.6 mg GAE 100 g^−1^), without significant differences among the 1‐MCP doses (*P* > 0.05) (Fig. 7(B)).

The low TPC content in untreated fruit was reflected in lower AOX values measured by both the ABTS and DPPH assays (*P* < 0.05). For the ABTS method and ‘Tommy Atkins’, AOX was 9.21 mM Trolox 100 g^−1^ at harvest. After cold storage, the AOX in untreated fruit was reduced to 2.70 mM Trolox 100 g^−1^, and 1‐MCP‐treated fruit retained higher AOX, especially at 600 nL L^−1^ (10.03 mM Trolox 100 g^−1^). After shelf life, untreated fruit had lower AOX (2.50 mM Trolox 100 g^−1^) than 1‐MCP‐treated fruit, especially at 600 nL L^−1^ (6.23 mM Trolox 100 g^−1^) (Fig. 7(C)).

A similar pattern was observed with ‘Keitt’ mango. The AOX was 8.14 mM Trolox 100 g^−1^ at harvest, decreasing to 1.43 mM Trolox 100 g^−1^ in untreated fruit after cold storage, whereas the 1‐MCP‐treated fruit retained a higher AOX (3.66–5.24 mM Trolox 100 g^−1^). After shelf life, untreated fruit had very low AOX (1.36 mM Trolox 100 g^−1^), differing significantly from all 1‐MCP treatments (2.56–3.24 mg TE 100 g^−1^), except the 600 nL L^−1^ dose (Fig. 7(D)).

For the DPPH method, ‘Tommy Atkins’ mangoes showed an AOX of 9.75 mg TE 100 g^−1^ at harvest. After 45 days of cold storage, the highest AOX levels were found in 1‐MCP‐treated fruit (7.85–8.99 mg TE 100 g^−1^), with no differences among doses, whereas untreated fruit showed a significantly lower value (4.68 mg TE 100 g^−1^). After shelf life, 1‐MCP at 400 nL L^−1^ resulted in the highest AOX values (8.64 mg TE 100 g^−1^), but did not differ from 600 nL L^−1^, and untreated fruit showed the lowest AOX (4.31 mg TE 100 g^−1^) (Fig. 7(E)).

For ‘Keitt’, a similar trend was observed with an AOX of 9.10 mg TE 100 g^−1^ at harvest. After 45 days of cold storage, 1‐MCP‐treated fruit exhibited the highest AOX values (7.17–8.52 mg TE 100 g^−1^), and untreated fruit had the lowest values (3.67 mg TE 100 g^−1^). After shelf life, 1‐MCP‐treated fruit had the highest AOX (5.92–7.35 mg TE 100 g^−1^), with no differences among doses, whereas untreated fruit had the lowest AOX (3.67 mg TE 100 g^−1^) (Fig. 7(F)).

### Experiment 3: validation of 1‐MCP for alleviating BF and preserving mango quality

According to the study described above, the 1‐MCP concentration of 200 nL L^−1^ was the lowest effective dose to inhibit BF incidence and reduce its severity, showing similar BF inhibition with higher doses. The 1‐MCP dose of 200 nL L^−1^ was therefore chosen to be validated in a new batch of ‘Tommy Atkins’ and ‘Keitt’ mangoes produced in the São Francisco Valley, Petrolina, PE, Brazil the following year.

For ‘Tommy Atkins’ mangoes stored at 9 °C for 45 days, 1‐MCP treatment reduced BF incidence from 45% to 20%, while severity decreased from 8.4% to 6.7% (Table 1). Following 7 days of shelf life, incidence was 26.6% in treated fruit compared to 66.6% in the control; similarly, severity was reduced to 7.0% from 36.2% (Table 1).

**Table 1:** Black flesh (BF) incidence and severity, and physiological attributes of untreated (0 nL L^−1^) and 1‐methylcyclopropene (1‐MCP)‐treated (200 nL L^−1^) ‘Tommy Atkins’ and ‘Keitt’ mangoes after 45 days of storage at 9 °C and 7 days of shelf life at 20 °C

Variable	At harvest	45 days of cold storage		45 days plus 7 days shelf life	
		Control	1‐MCP	Control	1‐MCP
Tommy Atkins					
BF incidence (% of fruit)	0 ± 0	45.0 ± 5.17 a*	20.0 ± 7.18 b	66.6 ± 18.86 a	26.6 ± 9.43 b
BF severity (% of flesh)	0 ± 0	8.4 ± 5.22 a	6.7 ± 2.22 b	36.2 ± 15.44 a	7.0 ± 6.00 b
Ethylene (nL kg^−1^ h^−1^)	0.02 ± 0.05	0.22 ± 0.12 a	0.00 ± 0.00 b	6.10 ± 4.70 a	0.50 ± 0.78 b
Respiration (mL CO₂ kg^−1^ h^−1^)	14.3 ± 1.70	21.5 ± 1.10 a	9.1 ± 0.60 b	54.5 ± 3.0 a	45.9 ± 0.9 b
Firmness (*N*)	55.4 ± 4.30	25.5 ± 2.50 b	30.5 ± 0.50 a	7.7 ± 3.7 b	14.2 ± 2.5 a
Peel color (°hue)	98.1 ± 3.30	83.5 ± 0.60 a	83.1 ± 2.30 a	82.6 ± 3.4 a	82.6 ± 3.5 a
Flesh color (°hue)	76.5 ± 1.80	77.3 ± 0.82 a	74.7 ± 0.60 b	75.3 ± 1.2 a	75.5 ± 0.7 a
SSC (%)	8.5 ± 0.40	10.3 ± 0.90 b	11.7 ± 0.40 a	10.0 ± 2.0 a	11.4 ± 0.6 a
TA (% citric acid)	1.80 ± 0.08	1.15 ± 0.03 b	1.23 ± 0.07 a	0.57 ± 0.19 a	0.65 ± 0.09 a
TPC (mg GAE 100 g^−1^ FW)	48.23 ± 0.66	22.76 ± 0.92 b	39.99 ± 1.19 a	7.97 ± 0.48 b	20.78 ± 0.72 a
ABTS (mg TE 100 g^−1^ FW)	10.18 ± 0.31	4.64 ± 0.73 b	8.23 ± 0.29 a	3.47 ± 0.32 b	6.48 ± 0.11 a
DPPH (mg TE 100 g^−1^ FW)	11.45 ± 0.40	6.23 ± 0.38 b	9.35 ± 0.43 a	4.87 ± 0.35 b	7.90 ± 0.16 a
Keitt					
BF incidence (% of fruit)	0 ± 0	100.0 ± 0.00 a	2.5 ± 2.00 b	100.0 ± 0.00 a	6.2 ± 5.22 b
BF severity (% of flesh)	0 ± 0	72.5 ± 7.72 a	3.7 ± 3.50 b	83.6 ± 13.14 a	1.8 ± 1.20 b
Ethylene (nL kg^−1^ h^−1^)	0.15 ± 0.06	15.50 ± 2.29 a	0.10 ± 0.08 b	18.40 ± 7.62 a	0.70 ± 0.36 b
Respiration (mL CO₂ kg^−1^ h^−1^)	21.3 ± 1.85	36.2 ± 2.80 a	23.4 ± 5.50 b	62.5 ± 8.9 a	46.8 ± 5.0 b
Firmness (N)	98.2 ± 8.40	2.2 ± 0.10 b	42.3 ± 16.70 a	2.3 ± 0.3 b	11.7 ± 4.5 a
Peel color (°hue)	103.4 ± 3.90	104.6 ± 5.70 b	111.5 ± 2.80 a	96.7 ± 5.1 b	108.2 ± 2.0 a
Flesh color (°hue)	81.3 ± 2.80	72.1 ± 5.00 b	78.0 ± 1.10 a	63.3 ± 5.2 b	77.1 ± 1.1 a
SSC (%)	8.2 ± 1.70	13.7 ± 0.90 a	13.5 ± 0.60 a	12.2 ± 0.5 b	14.0 ± 0.6 a
TA (% citric acid)	1.36 ± 0.11	0.96 ± 0.03 b	1.09 ± 0.04 a	0.88 ± 0.13 a	0.78 ± 0.13 a
TPC (mg GAE 100 g^−1^ FW)	46.91 ± 1.16	16.95 ± 0.36 b	33.38 ± 0.80 a	10.53 ± 0.17 b	16.58 ± 0.17 a
ABTS (mg TE 100 g^−1^ FW)	9.82 ± 0.25	2.86 ± 0.52 b	7.09 ± 0.55 a	2.24 ± 0.32 b	5.40 ± 0.22 a
DPPH (mg TE 100 g^−1^ FW)	10.19 ± 0.18	6.51 ± 0.40 b	10.93 ± 0.83 a	5.04 ± 0.08 b	7.91 ± 0.18 a

* Mean values followed by the same letter for each variable are statistically equal according to the least significant difference (LSD) test (*P* < 0.05). 1‐MCP = methylcyclopropene, BF = black flesh.

For ‘Keitt’ mangoes, the impact was more pronounced. After 45 days at 9 °C, 1‐MCP reduced BF incidence from 100% to 2.5% and severity from 72.5% to 3.7% (Table 1). After shelf life, treated fruit maintained a low incidence (6.2%) and severity (1.8%), whereas untreated fruit remained at 100% incidence and 83.6% severity (Table 1).

The application of 1‐MCP markedly suppressed ethylene production in both cultivars (Table 1). For ‘Tommy Atkins’, ethylene production by untreated fruit was 0.22 nL kg^−1^ h^−1^ after 45 days of cold storage, and treated fruit ethylene production was undetectable. After shelf life, ethylene production by untreated fruit increased to 6.10 nL kg^−1^ h^−1^, whereas treated fruit exhibited low ethylene production (0.50 nL kg^−1^ h^−1^). A similar response was observed with ‘Keitt’ mangoes, for which 1‐MCP reduced ethylene production from 15.50 to 0.10 nL kg^−1^ h^−1^ after storage, and from 18.40 to 0.70 nL kg^−1^ h^−1^ after shelf life (Table 1).

The respiration rate was also significantly reduced by 1‐MCP. For ‘Tommy Atkins’, respiration was decreased by 1‐MCP from 21.5 to 9.1 mL CO_2_ kg^−1^ h^−1^ after cold storage and from 54.5 to 45.9 mL CO_2_ kg^−1^ h^−1^ after shelf life. For ‘Keitt’, untreated fruit exhibited high respiration rates of 36.2 and 62.5 mL CO_2_ kg^−1^ h^−1^, whereas 1‐MCP‐treated fruit showed significantly lower rates of 23.4 and 46.8 mL CO_2_ kg^−1^ h^−1^ after storage and shelf life, respectively (Table 1).

Fruit firmness was significantly preserved by 1‐MCP application (*P* < 0.05). ‘Tommy Atkins’ mangoes had an initial firmness of 55.4 N at harvest. After cold storage, 1‐MCP‐treated fruit remained firmer (30.5 N) than untreated fruit (25.5 N), and this difference persisted after shelf life with values of 14.2 N for treated fruit and 7.7 N for untreated fruit. The effect was even more pronounced in ‘Keitt’, for which untreated fruit softened from 98.2 N at harvest to 2.2 N after cold storage and 2.3 N after shelf life, whereas 1‐MCP‐treated fruit maintained considerably higher flesh firmness of 42.3 N after cold storage and 11.7 N after shelf life (Table 1).

Peel color was not significantly affected by 1‐MCP for ‘Tommy Atkins’ at both evaluation periods. Conversely, for ‘Keitt’, 1‐MCP‐treated fruit maintained higher peel hue values (less yellow or orange), compared with untreated fruit, both after cold storage and shelf life. For flesh color, 1‐MCP preserved significantly higher hue values in ‘Keitt’, particularly after shelf life, compared with untreated fruit. In ‘Tommy Atkins’, differences in flesh color were less evident, with only a slight reduction observed after cold storage (74.7° in 1‐MCP treated fruit vs 77.3° in control fruit) (Table 1).

The SSC increased slightly in 1‐MCP‐treated ‘Tommy Atkins’ fruit, compared with untreated fruit after cold storage but no significant differences were observed after shelf life. For ‘Keitt’, SSC was unaffected by 1‐MCP after cold storage, but it was significantly higher in treated fruit after shelf life. The TA showed a modestly higher value in 1‐MCP‐treated ‘Tommy Atkins’ fruit after cold storage, compared with untreated fruit (1.23 vs 1.15%), whereas no differences were observed between treated and untreated fruit after shelf life. For ‘Keitt’, TA was slightly higher in 1‐MCP‐treated fruit after cold storage, compared with untreated fruit, but declined to similar levels to the control after shelf life (Table 1).

The preservation of bioactive compounds was strongly improved by 1‐MCP. At harvest, TPC values were 48.23 mg GAE 100 g^−1^ in ‘Tommy Atkins’ and 46.91 mg GAE 100 g^−1^ in ‘Keitt’ mangoes. The TPC values in ‘Tommy Atkins’ fruit were significantly higher with 1‐MCP, compared with control fruit, both after cold storage (39.99 vs 22.76 mg GAE 100 g^−1^) and after shelf life (20.78 vs 7.97 mg GAE 100 g^−1^). Similarly, for ‘Keitt’, TPC was maintained higher in 1‐MCP‐treated fruit compared with untreated fruit, both after cold storage and after shelf life (Table 1).

The AOX, measured by both ABTS and DPPH assays, was consistently higher in 1‐MCP‐treated fruit of both cultivars. For ‘Tommy Atkins’, AOX determined by the ABTS method was 10.18 mg TE 100 g^−1^ at harvest and was retained at 8.23 mg TE 100 g^−1^ after cold storage and 6.48 mg TE 100 g^−1^ after shelf life in 1‐MCP‐treated fruit. In contrast, untreated fruit showed a marked reduction in AOX, with values decreasing to 4.64 mg TE 100 g^−1^ after storage and 3.47 mg TE 100 g^−1^ after shelf life. A similar trend was observed for antioxidant activity (AOX) measured by DPPH. From an initial harvest value of 11.45 mg TE 100 g⁻¹, AOX decreased after cold storage to 9.35 mg TE 100 g⁻¹ in treated fruit compared with 6.23 mg TE 100 g⁻¹ in the control. Following shelf life, these values further declined to 7.90 and 4.87 mg TE 100 g⁻¹, respectively (Table 1).

For ‘Keitt’ mangoes, AOX measured by the ABTS method was 9.82 mg TE 100 g⁻¹ at harvest. Following cold storage, this value decreased slightly in 1‐MCP‐treated fruit (to 7.09 mg TE 100 g⁻¹) but fell sharply in untreated fruit (to 2.86 mg TE 100 g⁻¹). After shelf life, 1‐MCP‐treated fruit maintained higher AOX (5.40 mg TE 100 g⁻¹) than untreated fruit (2.24 mg TE 100 g⁻¹). A similar pattern occurred with the DPPH method; from a harvest value of 10.19 mg TE 100 g⁻¹, AOX after cold storage was 10.9 mg TE 100 g⁻¹ in 1‐MCP‐treated fruit compared with 6.5 mg TE 100 g⁻¹ in the control. After shelf life, these values were 7.91 and 5.04 mg TE 100 g⁻¹, respectively (Table 1).

## DISCUSSION

Black flesh (BF) is an internal physiological disorder in mango that remains poorly understood, despite its substantial impact on fruit quality and marketability. It is characterized by diffuse darkening of the fruit mesocarp, ranging from brown to black.^12^, ^13^ It is often confused with other internal disorders collectively classified as internal breakdown.^14^

Understanding the factors that contribute to BF incidence is crucial in order to develop effective mitigation strategies. Its subtle nature, with minimal external symptoms, hampers detection during commercial handling and underscores the need for deeper investigation into the mechanisms underlying its incidence and severity.^15^ The first reports of BF were documented in Mexico for ‘Haden’ mangoes cold stored for more than 20 days,^35^ and for ‘Haden’ fruit cold stored for more than 22 days.^36^ Recently, it has been suggested that BF could be a symptom of CI. This was based on observing it only after the fruit was stored at a temperature of 10 °C but not at 24 °C.^12^

To date, it has been unclear whether BF should be classified as a CI symptom.^12^, ^13^ The results of this study, however, indicate clearly that BF represents a symptom of CI strongly influenced by both storage temperature and harvest maturity. Evaluating three cultivars, ‘Tommy Atkins’, ‘Kent’, and ‘Palmer’, it was possible to determine that early harvested fruit consistently exhibited higher BF incidence than later harvested, half‐ripe fruit. This observation is consistent with much research showing that sensitivity to CI decreases as mango fruit mature and ripen.^37^, ^38^, ^39^ This study also showed that BF only occurred when fruit were stored at the lower temperatures of 8 and 10 °C, but no symptoms were observed at 12.5 °C in any cultivar or maturity stage, which is also consistent with many previous reports on effects of storage temperature on mango CI.^12^, ^14^ It appears that 12.5 °C may represent a safe threshold for preventing BF under the tested conditions. These findings agree with other studies that have reported BF symptoms in ‘Zill’ mangoes from China after storage for at least 20 days at 5 °C or 28 days at 10 °C, but not at 14 °C.^30^, ^31^

Recently, visible‐near infrared (visible–NIR) spectroscopy and machine learning‐based modeling have been proposed as alternatives for predicting the incidence of BF.^40^ However, effective strategies to prevent or reduce the BF disorder in mango remain limited. This study proposed, for the first time, the application of 1‐MCP as a potential postharvest intervention to reduce both BF incidence and severity in mango. The results demonstrated that 1‐MCP application was an effective strategy to reduce both the incidence and severity of BF in cold‐stored mangoes, as it has also been reported to inhibit CI symptoms in other species such as avocado,^23^ nectarine,^24^ and apple.^25^ In both ‘Tommy Atkins’ and ‘Keitt’, BF incidence reached 100% in untreated fruit after 45 days at 9 °C, while even the lowest 1‐MCP dose (200 nL L^−1^) reduced the disorder to below 60%. These findings are consistent with the previously known effects of 1‐MCP in alleviating CI‐related physiological disorders in mango.^11^

The beneficial effects of 1‐MCP are closely linked to its ability to suppress ethylene responses, such as ethylene production and respiration, which are key factors associated with CI susceptibility in banana and peach.^19^, ^41^ A meta‐analysis of 292 studies on climacteric fruits demonstrated that 1‐MCP treatments typically reduce internal ethylene concentration by 89%, ethylene production by 65%, and respiration rate by 25%,^42^ reflecting its role as an ethylene receptor antagonist and inhibitor of ethylene‐mediated signaling pathways.^43^, ^44^ In this study, 1‐MCP‐treated mangoes exhibited substantially lower ethylene production and respiration rates compared with untreated fruit, regardless of the dose. These findings align with reports on other tropical fruits, where 1‐MCP suppressed ripening‐related physiological parameters.^45^, ^46^ As 1‐MCP attenuated both ripening and BF development, the results of the experiments in this study suggest that BF may be associated with the ripening of mangoes exposed to chilling conditions.

The results also showed that 1‐MCP effectively delayed the softening of ‘Tommy Atkins’ and ‘Keitt’ mangoes, mainly during cold storage. Treated fruit maintained greater firmness in comparison with untreated fruit during storage. This result agrees with other studies using ‘Kensington Pride’ and ‘Tainong’ mangoes, both with 1000 nL L^−1^ of 1‐MCP.^27^, ^47^ The preservation of fruit firmness is likely related to the inhibition of ethylene signal transduction by 1‐MCP, which leads to cell wall breakdown.^19^ Ethylene stimulates the activity of cell wall‐degrading enzymes, including polygalacturonase (PG), pectin methylesterase (PME), and cellulases. In mango, 1‐MCP counteracts ethylene‐induced softening by modulating the expression of genes such as ethylene response factor (*MiERF*) and polygalacturonase (*MiPG*).^48^ By irreversibly binding to ethylene receptors, 1‐MCP blocks ethylene perception and signal transduction, thereby reducing the expression of genes involved in cell‐wall breakdown.^49^ However, both 1‐MCP treated and untreated fruit showed softening after transferring to shelf‐life conditions, reaching the ready‐to‐eat developmental stage, which is highly desirable to ensure fruit commercialization and consumption. These results suggest that the effect of 1‐MCP in maintaining fruit firmness decreased during shelf life. This is possibly related to higher ethylene receptor turnover and synthesis during ripening at the higher shelf‐life temperature, which resulted in greater fruit sensitivity to ethylene, as has also been reported in other studies.^50^, ^51^, ^52^, ^53^

As BF is an internal disorder, symptoms are typically restricted to the mesocarp and may not be externally visible.^14^ Consequently, changes in peel color are not reliable indicators of the presence of physiological disorders. In the flesh, however, fruit subjected to 1‐MCP application exhibited a higher hue angle, compared to untreated fruit, which agrees with the findings of previous studies with ‘Tommy Atkins’ mango.^54^

In this study, an absence of consistent 1‐MCP effects was found for SSC and TA. One of the key effects of physiological disorders on sugar metabolism is altered balance between starch and soluble sugars. Starch accumulates in healthy mangoes during fruit development and is subsequently converted into soluble sugars during ripening. However, physiological disorders can cause excessive premature degradation of starch into sugars, leading to an imbalance that might compromise the fruit taste and texture.^55^

Beyond its effects on the ripening physiology of ‘Tommy Atkins’ and ‘Keitt’ mangoes, 1‐MCP consistently maintained higher AOX and TPC in the fruit, compared with untreated fruit. It is known that CI in tropical fruits is closely linked to oxidative stress and the regulation of antioxidant metabolism.^56^ Chilling injury‐related disorders compromise fruit quality by inducing an imbalance between ROS accumulation and the fruit's antioxidant capacity. Elevated ROS levels, including MDA and hydrogen peroxide (H_2_O_2_), are recognized contributors to CI, as they can impair cell membranes and disrupt metabolic activities that are essential for fruit viability and quality.^49^, ^57^ As oxidative stress is a central component of CI, the maintenance of antioxidant metabolism by 1‐MCP may play a crucial role in inducing chilling tolerance,^58^ reducing BF development in mango.

Interestingly, the effect of 1‐MCP on BF in both ‘Tommy Atkins’ and ‘Keitt’ mangoes was not strictly dose‐dependent, as low and intermediate concentrations were generally as effective as higher doses in reducing the disorder. This indicates that low 1‐MCP concentrations (200 nL L^−1^) may be sufficient to achieve meaningful reductions in BF incidence and severity, which has practical implications for postharvest management strategies. Taken together, these findings indicate that 1‐MCP has strong potential as a postharvest treatment to mitigate BF in mangoes, primarily by suppressing ethylene‐mediated ripening processes and modulating AOX. However, given that the efficacy of 1‐MCP may depend strongly on the cultivar, harvest maturity stage, and storage conditions,^42^ further studies are warranted to optimize dose, timing, and integration with other postharvest technologies to ensure consistent efficacy across different production systems.

**Figure 8:**
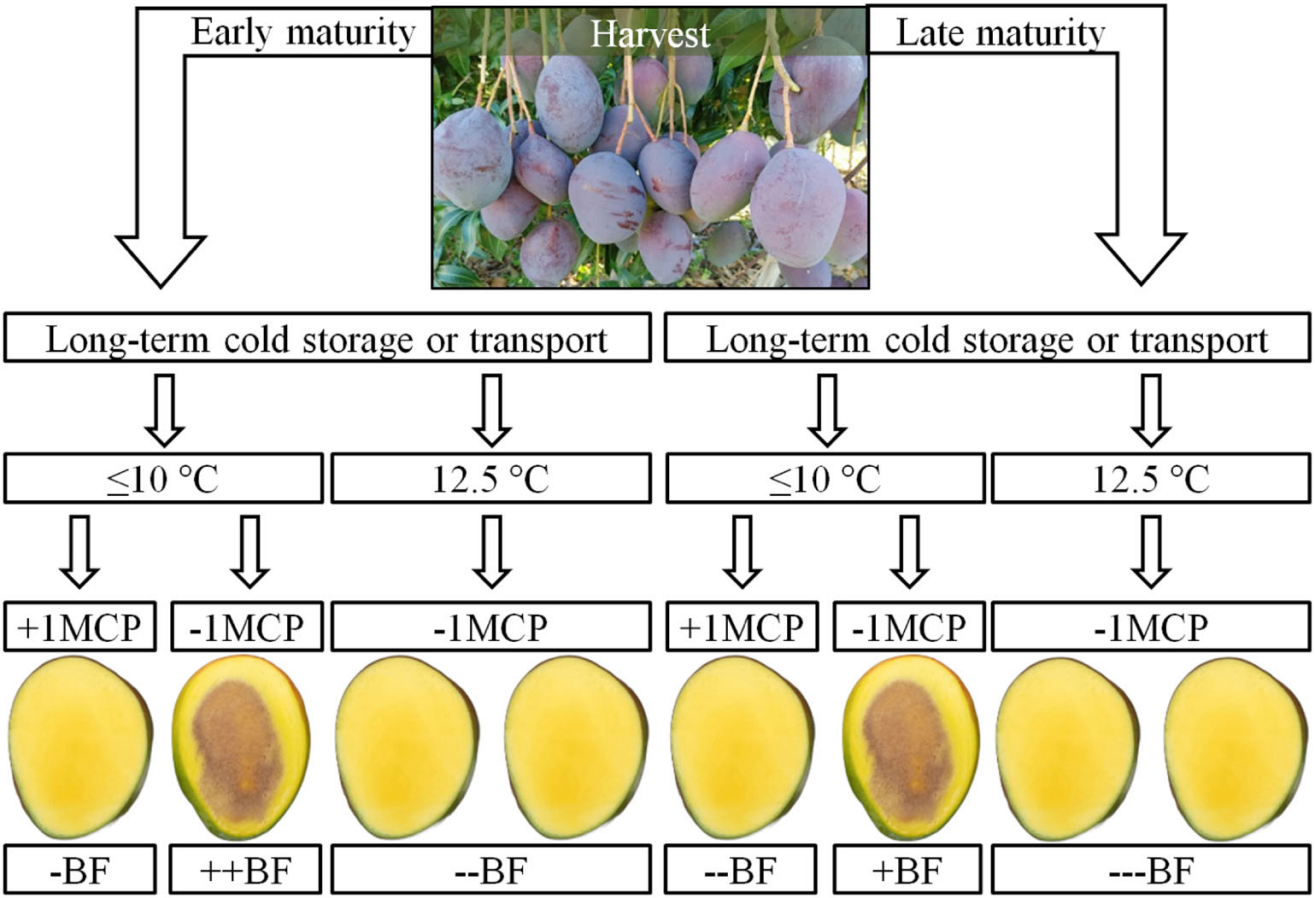
Schematic representation illustrating the probability of black flesh (BF) incidence in mangoes during long‐term storage or transportation as influenced by harvest maturity, storage temperature, and 1‐methylcyclopropene (1‐MCP) treatment. Probability of BF incidence was represented as very high (++BF) to very low (−BF).

Considering the probability of BF incidence in fruit from orchards with a history of high BF occurrence, this study indicated that mangoes harvested at early maturity and stored at low temperatures (≤10 °C) without 1‐MCP treatment exhibited the highest probability of BF incidence (++BF). However, this incidence could be markedly reduced by 1‐MCP application (−BF) or by storing the fruit at a higher temperature of 12.5 °C (−BF) (Fig. 8). Conversely, harvesting fruit at more advanced maturity stages tended to decrease BF incidence in untreated fruit (+BF) and enhance the suppressive effects of both 1‐MCP treatment (−BF) and higher storage temperature (12.5 °C; −BF) on BF incidence during long‐term cold storage or transportation (Fig. 8).

## CONCLUSION

Black flesh is a CI symptom that is strongly aggravated by early harvest and storage at or below 10 °C, whereas storage at 12.5 °C completely prevents the disorder in all evaluated mango cultivars.

The application of 1‐MCP mitigated BF incidence and severity in both ‘Tommy Atkins’ and ‘Keitt’ mangoes, suppressing ethylene production, reducing respiration rate, maintaining flesh firmness, and preserving the antioxidant system during cold storage and subsequent shelf life.

Among the 1‐MCP concentrations tested, 200 nL L^−1^ proved sufficient to reduce BF incidence and severity, and to maintain higher flesh firmness during long‐term storage.

1‐Methylcyclopropene represents a promising postharvest strategy to alleviate chilling‐related disorders and extend the postharvest life of mangoes without compromising fruit quality.

## AUTHOR CONTRIBUTIONS

**Bruna Parente de Carvalho Pires:** writing – review and editing, writing – original draft, visualization, validation, methodology, investigation, formal analysis, data curation, conceptualization. **João Claudio Vilvert:** writing – review and editing, writing – original draft, visualization, validation, methodology, investigation, formal analysis, data curation, conceptualization. **Nilo Ricardo Corrêa de Mello Júnior:** validation, methodology, investigation, formal analysis. **Sandy Raiele Sena Monteiro:** validation, methodology, investigation, formal analysis. **Mikaele de Souza Santos:** validation, methodology, investigation, formal analysis. **Ester Silva Regis:** validation, methodology, investigation, formal analysis. **Jeffrey K. Brecht:** writing – review and editing, writing – original draft, visualization, methodology, conceptualization. **Sergio Tonetto de Freitas:** writing – review and editing, writing – original draft, visualization, validation, supervision, resources, project administration, methodology, investigation, funding acquisition, data curation, conceptualization. **Jackson Teixeira Lobo:** validation, methodology, investigation.

## CONFLICT OF INTEREST

The authors declare no conflict of interest.

## Data Availability

Data will be made available on request.
